# Policy Initiatives Regarding the Long-Term Care Immigrant Workforce and Associated Outcomes: A Scoping Review

**DOI:** 10.1093/geroni/igaf122.1835

**Published:** 2025-12-31

**Authors:** Moroni Fernandez Cajavilca, Lei Chen

**Affiliations:** University of Utah, Salt Lake City, Utah, United States; University of California, San Francisco, San Francisco, California, United States

## Abstract

It is widely acknowledged that a significant shortage of nurses and direct care workers willing to work with older adults in long-term care (LTC) settings exists. The LTC industry has addressed this issue by advocating for expanding the immigrant labor force to strengthen the nursing workforce. However, there has been insufficient attention to immigration reform that could optimize the contributions of immigrant workers in the LTC sector while protecting their health and well-being, which may impact the care they provide. This scoping review examined how policy initiatives, or lack thereof, influence retention, protection, and/or health outcomes of the LTC immigrant workforce. We conducted a literature search with a librarian to tailor searches for five databases: Medline, CINAHL, ABI/INFORM, PAIS Index, and Web of Science. Inclusion criteria included articles globally and between 2010-2024 that focused on national, state, or organizational policy initiatives related to the LTC immigrant workforce and/or their health outcomes. We used Covidence, which automatically excluded duplicates (n = 387). Two reviewers screened by text and abstract (n = 1149), followed by full-text screening (n = 113). We identified 46 articles that met our inclusion criteria. The findings suggest there is a dearth of policy initiatives designed to retain or protect the LTC immigrant workforce. Moreover, the health outcomes of the LTC immigrant workforce are generally negative, owing to the absence of comprehensive policy initiatives. Studies largely reported the need for specific policy initiatives (e.g., language access, payment restructures, antiracism policies), which may be innovative approaches to encouraging, supporting, and fostering the LTC immigrant workforce.

